# Performance evaluation of reduced complexity deep neural networks

**DOI:** 10.1371/journal.pone.0319859

**Published:** 2025-03-20

**Authors:** Shahrukh Agha, Sajid Nazir, Mohammad Kaleem, Faisal Najeeb, Rehab Talat

**Affiliations:** 1 Department of Electrical and Computer Engineering, COMSATS University, Islamabad, Pakistan; 2 School of Computing, Engineering and Built Environment, Glasgow Caledonian University, Glasgow, Scotland, United Kingdom; 3 Islamic International Medical College, Riphah International University, Rawalpindi, Pakistan; Commonwealth Scientific and Industrial Research Organisation, AUSTRALIA

## Abstract

Deep Neural Networks (DNN) have achieved state-of-the-art performance in medical image classification and are increasingly being used for disease diagnosis. However, these models are quite complex and that necessitates the need to reduce the model complexity for their use in low-power edge applications that are becoming common. The model complexity reduction techniques in most cases comprise of time-consuming operations and are often associated with a loss of model performance in proportion to the model size reduction. In this paper, we propose a simplified model complexity reduction technique based on reducing the number of channels for any DNN and demonstrate the complexity reduction approaches for the ResNet-50 model integration in low-power devices. The model performance of the proposed models was evaluated for multiclass classification of CXR images, as normal, pneumonia, and COVID-19 classes. We demonstrate successive size reductions down to 75%, 87%, and 93% reduction with an acceptable classification performance reduction of 0.5%, 0.5%, and 0.8% respectively. We also provide the results for the model generalization, and visualization with Grad-CAM at an acceptable performance and interpretable level. In addition, a theoretical VLSI architecture for the best performing architecture has been presented.

## 1. Introduction

Deep Neural Networks (DNN) are used for segmentation and classification tasks in medical imaging and the results can often exceed those of experienced medical practitioners. Although DNNs provide very good performance but comprise of very compute intensive processing. This complexity prevents its use in mobile and other embedded platforms specifically where a quick inference is required, such as, in healthcare applications. There are many approaches that are used to reduce the DNN model complexity while aiming for as little accompanying model performance degradation as possible. Quantization of the model parameters to integers is associated with a corresponding loss in model’s performance [1]. Besides quantization, network pruning is another promising technique [2–4]. The number of model parameters can also be decreased through reducing the model’s computational complexity [5].

The DNN model after being trained on an annotated dataset can be used on an unseen dataset to determine the model performance. In contrast, transfer learning uses a pre-trained model as a classifier for another dataset by re-training some of the later layers. This exploits the features that were learnt by the model and can significantly decrease the training time and cost. Transfer learning was used to determine the severity grading with opacity score and geographic extent for positive COVID-19 patients, for a limited dataset using a VGG16 model [6]. Although the peak of the COVID-19 has passed, but a danger of its variants reaching a pandemic state still exists. COVID-19 has resulted in 704,645,434 cases and 7,009,825 deaths worldwide to date [7]. Several pre-trained Convolutional Neural Network (CNN) models were used for binary and multiclass classification into normal, viral, and COVID-19 [8]. The COVID-19 differentiation from pneumonia and lung cancer, and the severity classification of COVID-19 is hard [8]. The CXR images of viral pneumonia are hard to be differentiated by radiologists from COVID-19 as these lung diseases are manifested with lung abnormalities that overlap [9]. For CXR images the interclass similarity for COVID-19, bacterial and viral pneumonia can be quite high, making it a challenging task for radiologists [10]. Model generalization means that the trained DNN model has learnt features such that it can provide a similar performance on another dataset that might be from a different site. The differences between the datasets exist due to the difference in the image acquisition protocols, medical imaging equipment, and the operator expertise and training. It is thus important that a trained DNN can generalize to the other similar datasets.

DNN models although useful for disease diagnosis but are like a black-box reducing the accompanying trust in the automated system due to a lack of understanding by a human as to why a particular model decision was made. This has been addressed by the application of Explainable AI techniques, that can not only help to understand why a particular decision was made, but can also help support or negate a radiologist’s decision, help improve a model, and to find instances where the model is making a correct prediction decision but for a wrong reason [11]. Gradient-weighted Class Activation Mapping (Grad-CAM) is one such widely used technique that helps by highlighting the image areas considered important by a model using a heatmap [12]. The use of Grad-CAM can help a radiologist in improving their decision in identifying a positive case of pneumonia or COVID-19. Some studies have used lung segmentation prior to classification providing an advantage that the irrelevant features are segregated and location-specific features are extracted, improving the classification reliability [13]. Infection maps generation was proposed through a joint localisation, detection and severity grading of COVID-19 in CXR images [14]. The limited dataset size was addressed by proposing a patch-based CNN and using majority voting for the final decision [15]. The interpretability of the predicted images was demonstrated with changes to Grad-CAM [15].

In [16], a transfer learning based technique was utilized for high precision underwater image classification using EfficientB0 pretrained on ImageNet dataset. This neural network was then combined with two-hidden-layer random vector functional link network to extract the features from underwater images to train the network on underwater image dataset. The authors emphasized the importance of using two hidden layers with same number of nodes for to achieve a high precision, and reported 87.28%, 74.06%, and 99.59% accuracy on the MLC2008, MLC2009, and Fish-gres datasets, respectively. Similarly, in [17] authors proposed a transfer learning based approach to train the neural network for the purpose of fish classification. They utilized ResNet50 pretrained on ImageNet dataset and avoided model overfitting and accuracy saturation. They utilized error-minimized random vector functional link as a classifier in place of Softmax. The authors reported accuracy rates reaching 99.68%, 97.34%, and 99.77% for the datasets URPC, LifeCLEF 2015, and Fish4Knowledge respectively. In [18], an automatic fabric wrinkle grading model based on an improved African Vultures Optimization (AVO) algorithm was proposed which optimized a regularized random vector functional link (RRVFL) network. Harris Hawks Optimization (HHO) algorithm was utilized for enhancing the model’s convergence speed and classification accuracy. Authors demonstrated that HHO-AVO-RRVFL model achieved an average classification accuracy of 97.86% across multiple datasets.

This work aims to address the following challenges:

- How to improve the classification accuracy: The authors have shown the efficacy of the proposed methodology in terms of metrics such as accuracy, recall, precision, f1-score, confusion matrix, ROC curves and Grad-CAM in Section 5.
- How to reduce the computational complexity of the neural network for edge devices with comparable quality: The authors have proposed a number of architectures with reduced computational complexity in Section 4.2.
- How to make the inference in minimal amount of time, i.e., in near-real time: There are many applications that require fast classification results, e.g., COVID detection on the spot using low power portable devices. For this purpose, authors have proposed a theoretical VLSI architecture that employs techniques such as parallelism, pipelining, retiming, architecture reusing and scalability etc. to achieve this aim. This has been mentioned in Section 5.6.

In this study, we aim to investigate the model complexity reduction for model adaptation towards edge computing and Very Large-Scale Integration (VLSI) implementation. For the model evaluation, we chose to use the CXR images for multiclass classification of the COVID-19, and pneumonia classes. We also investigate the model generalization using a different dataset and visualization using Grad-CAM technique.

The research contributions of this paper are:

- A low complexity ResNet-50 architecture for multiclass classification is proposed.
- Designing and comparing the complexity reduction choices with the original ResNet-50 model training using transfer learning and training from scratch, and proposed modified low complexity model training from scratch.
- Performance evaluation with complexity reduction of the proposed reduced complexity models for the multiclass classification and model generalization task on chest X-ray (CXR) images.
- Utilizing the visualization technique Grad-CAM for understanding the model focus area in the image as a means to augment the medical practitioner’s decision.
- A theoretical VLSI architecture of the best performing CNN is presented.

Rest of this paper is organized as follows: The related work is presented in Section 2. Section 3 describes the methodology. The results are presented in Section 4. Section 5 concludes the paper.

## 2. Related work

This section describes the research studies focussing on DNN model complexity reduction with a summary provided in Table 1. Parameter reduction using sparsity regularized factorization neurons for DNN models was proposed with an aim to minimize the classification errors [1]. It was reported that the parameter controlling the appropriate rank and the model reduction rate had issues with optimal value selection [1]. Pruning factors of up to 16 times were investigated using ResNet model without significant accuracy drop by the proposed budgeted regularized pruning framework [3]. A method for complexity reduction was proposed based on network quantization and out-in channel pruning [19]. The redundant out-in channels were recovered using a greedy pruning algorithm, and an incremental quantization algorithm for to smooth network fluctuations were used [19]. With ResNet-50 on ImageNet-1k dataset, a reduction of 50% Floating-point Operations Per Second (FLOPS) with 0.37% drop in accuracy was obtained [19]. The ResNet-50 model latency was shown to meet the chosen 34ms constraint with 0.82% of drop in accuracy through the use of a proposed latency predictor and a ZeroBN algorithm [2]. The authors demonstrated accuracy improvement of 0.24% to 0.32% on CIFAR-10 dataset.

**Table 1:** Related work on DNN complexity reduction.

DNN Model	Dataset	Application	Focus	Ref
Model agnostic	Speech corpus	Acoustic model	Parameter reduction using sparsity regularized factorization neurons	[1]
Model agnostic	CIFAR-10, ImageNet-100	Edge systems	DNNs under a ‘hard’ latency constraint upon targeted hardware by dynamically zeroizing and recovering Batch Normalization (BN) layers.	[2]
Model agnostic	–	Edge computing	General methods for complexity reduction	[20]
AlexNet, VGG-16, ResNet56	CIFAR-10, ImageNet	Model compression in seconds/minutes	Considering the whole network layers for accuracy/low rank kernel decomposition/computational complexity trade-off-layer wise accuracy metrics	[5]
Model agnostic-ResNets- Sec 2-previous works	CIFAR-10, CIFAR-100, MIO-TCD	Model accuracy	Structured pruning with budget aware regularization	[3]
AlexNet on Raspberry Pi	CIFAR-10, Network trace dataset	Mobile devices for 5G	Device-edge synergy for early inference	[23]
Model agnostic-VGG-16	MNIST, CIFAR-10, ImageNet, PASCAL VOC	Overall speedup	1D convolution using Toom-Cook algorithm	[22]
Model agnostic	MNIST, SVHN and NORB	Layer reduction factors	Non-uniform allocation of reduction factors to layers- pruning and low-rank approximation	[4]
Model agnostic	Wall Street Journal (WSJ0) dataset -50%reduction in DNN memory footprint while STOI (intelligibility) drops by 2.7%	Speech enhancement application	Non-uniform quantization of DNN parameters for different layers	[24]
AlexNet, ResNet-50, DenseNet, CifarNet	CIFAR-10/100, ImageNet-1K	Classification and style transfer tasks	Pruning and quantization	[19]
CNN, KFC, Dilated CNN	MNIST and Fashion MNIST		Parameter reduction via product-of-sums matrix decompositions	[21]

Optimization methods were proposed for introducing the model sparsity to reduce the model complexity with an aim to preserve the speed and accuracy, with accuracy reduction within 2%, for suitability on edge devices [20]. AlexNet, VGG-16, and ResNet-56 were used for evaluation of the proposed method for rank configuration which provided model compression in seconds or minutes compared to hours in the other selected models, while providing comparable accuracy [5]. For VGG-16 the reductions in FLOPs were reduced by 25% and accuracy was improved by 0.7% [5]. The model parameter reduction method was proposed using product-of-sums matrix decompositions demonstrating good accuracy with a small number of model parameters [21]. The model parameters for a CNN architecture were reduced from 3x10^6^ trainable parameters to only 3554 parameters obtaining 98.44% accuracy [21]. Significant model speedups were obtained through implementation of the fundamental convolution by Toom-Cook algorithm and modifying the convolutional layer structure achieving up to 10 times increase over the baseline method [22].

A layer wise reduction method for each layer was proposed to determine the optimal non-uniform allocation of the layer reduction factors to different layers to reduce the effect on the reduced model performance [4]. A non-uniform quantization approach was proposed to dynamically quantize the different layer parameters within the same layer for Field Programmable Gate Array (FPGA) applications [24]. The proposed method was evaluated on a speech dataset and showed a 50% reduction of the DNN memory with a performance degradation of only 2.7% [24]. In [25], authors conducted a study to investigate best performing neural network out of DarkNet-19, ResNet-101, SqueezeNet, VGG-16, and VGG-19, for the purpose of COVID-19 detection on chest X-ray images. Similarly, authors in [26] investigated Xception, DenseNet201, ResNet152V2, InceptionResNetV2, NASNetLarge, and VGG16 neural networks for the purpose of pneumonia detection through chest X-ray images. They trained and fine-tuned their chosen models with Nesterov Stochastic Gradient Descent optimizer with their respective learning rate.

In contrast to these studies, our proposed method is based on a simplified approach, and can be applied across any CNN model, and does not result in any significant performance loss considering the reduction in the model size.

## 3. Motivation

The idea in the manuscript stems from the fact that regular and efficient VLSI implementation results when the algorithm is equally regular and simple [27]. Authors have presented and added a theoretical VLSI architecture in section 5.6, which can be used for practical VLSI implementation, which has hardware efficiency [28] and data and architecture reuse. The idea of the present work stems from Baek et al. work [29], in which they proposed low power, low cost, and high speed VSLI architecture for reduced resolution motion estimation process. It was suggested to use only upper four bits of 8-bit pixel in the sum of absolute difference metric as the sum corresponding to lower four bits has normal distribution and hence its expected value is zero according to central limit theorem [29]. Shahrukh et al., then proposed [30], a correction mechanism for compensating the reconstruction error introduced due to ignoring lower four bits, as there were few image sequences which showed high error because of ignoring lower four bits. The correction unit is triggered conditionally when a certain condition arises, leading to the power efficient VLSI architecture with high accuracy. In the same way, in the present work, channels of the CNN have been reduced through channel pruning. This means number of filters have been reduced which reduces the number of weights.

For sequential implementation, i.e., either processor based or rolled VLSI based implementation, the number of computations has been reduced, leading to comparatively low inference time and low cost of the design. In this case more memory will be used for storing the weight coefficients and smaller arithmetic logic unit will suffice. On the other hand, if parallel VLSI implementation is used then it leads to very high throughput at a cost of more resources or logic as more multipliers and adders will be used. The following power consumption formula for the CMOS technology [28] explains this fact, i.e.,


$$P=CV^{2}f\alpha$$ (1)

where P is the power consumption, C is the total capacitance of the design, V is the applied voltage, f is the frequency of operation and α is the logic low to high average transitions per clock cycle.

As can be seen, high value of C leads to higher power consumption. On the other hand, frequency f can be varied to keep the power consumption at the required level by sacrificing speed of the design, or sacrificing power saving for high speed. The resulting error due to channel pruning is then compensated through retraining and fine tuning the CNN at low learning rate. The other way for reducing the cost of the VLSI architecture of CNN is to quantize the weights.

Through experimentation on COVID chest X-ray dataset and CIFAR-10 dataset, the authors concluded that the architecture 4 (Arch4), is more suitable for future VLSI implementation as it has same number of channels across the layers, leading to high data and architecture reuse, has comparatively reduced number of weights and almost the same accuracy level as that of the full channel CNN.

The idea in this work is to carry out the COVID detection in real time, i.e., the patient gets detected for COVID within two seconds after passing a digital X-ray scanner. In order to achieve this aim, low power and portable implementation of the CNN becomes mandatory. Though practical implementation is not presented in this work, however a theoretical idea for achieving this aim has been given through an efficient VLSI architecture.

## 4. Methodology

This section describes the CNN architecture and the proposed complexity reduction techniques, datasets used in the study, image classification, generalization, and explainability.

### 4.1. CNN architecture

Convolution neural network consists of number of interconnected layers. These layers are responsible for the computation of two dimensional (2D) discrete convolution, batch normalization, activation, pooling and zero padding function operations [31–33].

An m×n 2D discrete Convolution operation, which is a filtering operation, is mathematically defined as,


$$C\left(p,q\right)=\overset{m-1}{\underset{k_{1}=0}{\sum }}\overset{n-1}{\underset{k_{2}=0}{\sum }}img(p-k_{1},q-k_{2}).w\left(k_{1},k_{2}\right)$$ (2)

where $\left(p,q\right)$ are the coordinates of the image, img, where convolution operation is applied and *w* are the adjustable weight coefficients of the CNN. In practice the following convolution or correlation operation is applied which is obtained by mapping the 2D matrix of weight coefficients on the image portion and applying the dot product operation, i.e., mathematically,


$$C\left(p,q\right)=\overset{m-1}{\underset{k_{1}=0}{\sum }}\overset{n-1}{\underset{k_{2}=0}{\sum }}img(p+k_{1},q+k_{2}).w\left(k_{1},k_{2}\right)$$ (3)

where, $\left(p,q\right)$, in this case are the coordinates of the upper left corner of the image block. Indexes here are increasing from upper left corner to bottom right corner of the block. There are number of convolution layers in CNN with a purpose to extract features from the input image. For example, the starting layers extract simple features or low-level features from the input image such as curves and edges. The subsequent layers extract more complex features by subsampling and adding the features from the previous layers. In this manner when the final layer is reached, more complex features are extracted which help in determining the distinguishing features of the image or its overall definition.

Batch normalization refers to the process of making the distribution of feature vectors, zero mean and unity variance [31–33]. The application of batch normalization reduces the problem of overfitting, vanishing and exploding gradients and exploding feature values by constraining the feature values to the same scale, adding noise, making the optimization landscape smoother and making sure that no feature gets more priority than the other in making the decision at the output [31]. Batch normalization also accelerates the learning process as the distribution of features do not vary significantly, leading to an easy and fast learning process. The purpose of pooling layer is to reduce the redundancy in the features by subsampling it, leading to reduced computational complexity and prevention of overfitting.

Activation functions are used to introduce non-linearity in the network so that the network could approximate non-linear functions [31–33]. Rectified linear unit (ReLU) [31] is considered to be most suitable for CNN, due to its ability to prevent vanishing gradient problem during training, as this function helps to prevent the saturation of the neurons.

In CNN, a single 2D m×n convolution filter has m×n+1 learnable parameters, where m×n parameters are its weights, that are multiplied with the features from the previous layer and the last parameter is the bias, that is added to the result of convolution. A convolution operation can be strided, with stride length greater than one. Due to this the resolution of the output feature can be adjusted. The more the stride, the lower is the resolution. In strided operation, instead of sliding at each coordinate of feature or input image, the convolution operation computes at those locations that are separated by a stride length, both horizontally and vertically. Similarly, zero padding is used to extend the size of output feature such that its size is same as that of the input.

In batch normalization, the normalization is done over the batch of input images [31–33]. Just like convolution operation, batch normalization also has two learnable parameters *β* and *γ*, that are updated with each batch, where *β* scales the feature $x_{iB}$ and *γ* adds into it [31–33].

The weights and biases of the CNN are updated starting from the last layer and moving towards the initial layers. This process is known as backpropagation. In this process the gradients of loss function with respect to the weights of the layers, is calculated using chain rule. Optimization algorithms are utilized to minimize the loss function and update the weights and biases. One of the most common optimization algorithm is the stochastic gradient descent (SGD) algorithm that updates the weights and biases depending upon the value of gradients of the loss function. Other methods include Adam optimizer algorithm [31–33] which is used in this work.

It needs to be mentioned here that in batch stochastic gradient descent algorithm, the weights and biases are updated once per batch, i.e., for each input image, the gradient of loss function is computed. Then the average of all these gradients is computed per batch and the weights are updated. This batch SGD algorithm has added benefit of utilizing the overall error function [31–33]. Adam optimization algorithm is a combination of momentum and AdaGrad optimization algorithms and has more possibility of reaching the global minima by avoiding the local minima.

At the end of the CNN, there is a flattening of the output features, i.e., the multidimensional features are converted to a one-dimensional array. This array of features is then passed to fully connected (FC) layers. The size and number of such layers depends upon the architecture. FC layer consists of neurons, each neuron receives inputs from the previous layer multiplied by an adjustable weight parameter. Just like batch normalization, regularization of data output from the FC layer is done using $L_{1}$ and $L_{2}$ regularization. The purpose of this regularization is to prevent model overfitting.

The regularization procedure introduces a penalty term in the loss function, whose purpose is to make the weights simple or small. Prior to last FC layer, the intermediate FC layers usually use ReLU as activation function. However, the last convolution layer uses Softmax activation function [31] in order to output probability distribution over the classes.

It needs to be mentioned here that no dropout layer has been used with FC layers as implementing the dropout mechanism did not give any significant improvement. Instead $L_{1}$ and $L_{2}$ regularizations were utilized to prevent model overfitting.

### 4.2. Proposed complexity reduction

#### 4.2.1. ResNet-50.

**Fig 1:**
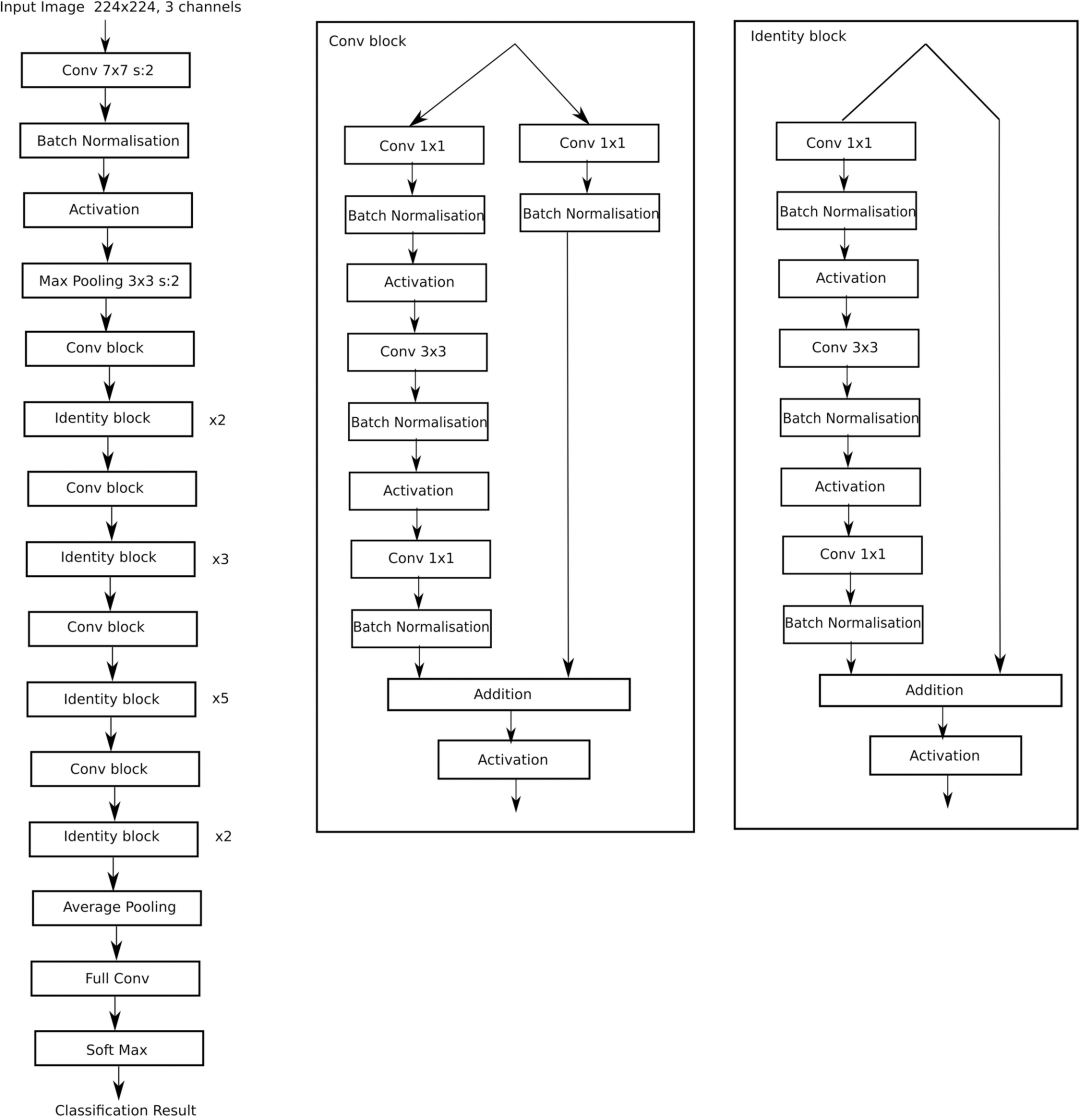
ResNet-50 architecture.

ResNet-50 model [32] was selected for DNN complexity reduction. ResNet-50 has been widely used in medical image classification and has shown better performance compared to many other neural networks [31–38]. ResNet-50 architecture [32] is a CNN which was introduced to circumvent the vanishing gradient problem. ResNet-50 achieved this by adding the original input to the output of blocks. This architecture consists of multiple residual blocks each block consisting of multiple convolution layers and a bypass connection from the input to output of the block, where the input of the blocks gets added to the output of the block. ResNet-50 architecture is shown in Fig 1.

Overall ResNet-50 architecture has 50 layers. It needs to be mentioned here that every convolution layer has a corresponding batch normalization layer and activation layer, at the output, not mentioned in the architecture above.

In convolution layers, the kernel or weight initializer used is “He Normal” [31]. It draws samples from a truncated normal distribution centered on 0 with standard deviation equal to $\sqrt{\frac{2}{fan\_in}}$ where fan_in is the number of input units in the weight tensor. Weight initializing has its importance in the training performance of CNN and is linked to problems of vanishing and exploding gradients in CNN. The loss function utilized is categorical cross entropy [31] for multiclass classification.

One of the reasons for choosing ResNet-50 architecture in this work is the low error associated with it. For example, in [33], authors have mentioned that ResNet has the least top-1 and top-5 error as compared to LeNet, AlexNet, VGG and InceptionNet networks. Similarly, in [34], ResNet-50 has the highest accuracy when compared to LeNet-5, VGG-11, VGG-16 and VGG-11M networks. In [35], authors have reported that ResNet-50 network has less validation error than DenseNet-121, DenseNet-169 and ResNet-34 at a cost of a greater number of parameters involved. In [36], authors have mentioned the benefit of ResNet-50 network over XNOR-Net. In [37], authors have reported greater top-1 accuracy of ResNet-50 network, for ImageNet database, as compared to SqueezeNet, AlexNet, GoogleNet, ShuffleNet, ResNet-18, VGG16, VGG19, MobileNet v2 and NASNetMobile networks. In [38], it was reported that ResNet-50 network has better performance than VGG-16 and SqueezeNet neural networks.

It is important to mention here that other CNNs such as EfficientNet-B4 and Xception networks were also evaluated, however the training performance was almost same as that of ResNet-50.

#### 4.2.2. Model complexity reduction.

In CNN, each layer has number of filters to be applied to the preceding layer output. For example, if the first layer, layer1, of CNN is a convolution layer having *N* number of filters and input as image, then there will be *N* channels or feature maps at the output of this layer, each channel corresponding to the application of the corresponding convolution operation on the input image. Now if the succeeding convolution layer, layer2, has *M* number of filters, then implicitly, this layer has M×N number of filters and *M* number of output channels. In other words, this layer2 has two dimensional M×N matrix of points, each point corresponds to a different m×n filter. The first channel of the layer2 is obtained by convolving (using Eq. 3) the *N* channels of layer1 with *N* filters from the first row of M×N matrix and adding the results of the convolution operations. Similarly, other channels of layer2 will be obtained. This process will be repeated for the succeeding convolution layers.

It can be seen from Eq. 3 that a single convolution operation has m×n number of multiplications and m×n−1 number of additions. Increasing the image dimensions, number of channels, number of layers and filter size, leads to increased computational complexity of the CNN.

Reducing number of parameters of a network can lead to increased accuracy as it has the effect of preventing the network from overfitting just as is the effect of dropout layer. As is shown in the results section, reducing the number of features or channels up to some extent has actually increased the validation accuracy and generalization capability of the network. Reducing the network parameters also leads to an efficient VLSI implementation of a network with reduced area and power, and fast speed. However too much reduction of features is not beneficial.

In order to induce sparsity in deep neural network’s connection matrices, pruning can be used, leading to reduced computational complexity of the network [39]. In [40,41], authors reported that there is only a marginal loss in accuracy due to pruning, which on the other hand significantly reduces network complexity. In [39], pruned models (large-sparse) and small-dense models are compared. Both have approximately same number of parameters. It was found that large sparse models outperformed small-dense models in accuracy and achieved 10x reduction of non-zero parameters.

Accuracy performance of a network depends how deep and big neural network is. However, utilizing this network in resource constrained environments is quite challenging. It is not surprising for these models to exhibit on the order of billions of memory accesses and arithmetic operations in inference mode, making the process infeasible for real time processing, in addition it can easily drain the battery of the device and lead to heat dissipation. As an example, VGG16 model [42] has 138.34 million parameters which in turn requires 30.94 billion floating point operations to recognize a single 224x224 image. In [41,43], magnitude-based weight pruning methods were introduced in which filters with smallest weight magnitudes are eliminated. Apart from pruning, in [44], quantization-based technique was introduced which reduces the bitwidth of parameter from 32 to 8-bits. Pruning can be combined with quantization to achieve maximal compression. In [39], pruning is done after the model has been initiated for few epochs, or the model is pretrained. In [39], MobileNets are used for the comparison purpose between sparse and dense MobileNets. MobileNets are based on factorized convolutions known as depth wise separable convolutions which have reduced complexity as compared to standard convolution.

In [45], greedy driven filter pruning (GDFP) algorithm is mentioned, for model compression, which removes redundant weights while minimally impacting the accuracy. The criteria used for filter pruning is L_1_ norm of the kernel. For multichannel, the sum of L_1_ norm across all the channels is used to remove the unimportant filter. Overall, the complete model is trained first and then pruned and then again trained.

In this work, a similar approach to filter pruning, i.e., channel pruning is utilized. However, there is no criteria for reducing the number of channels. The reason is that by fixing the number of channels at compile time, the model is trained once and then evaluated. However, in case of filter pruning, as mentioned above, the network is trained twice.

One of the aims of the present work is to propose a low complexity network with acceptable accuracy level for resource constrained devices. In [46], authors have utilized Raspberry Pi 4 processor system to deploy their proposed low complexity network. Similarly, in [47], authors have used Winograd algorithm which reduces significantly the multiplication operations of convolution operation.

Keeping this in view, the three network architectures, Arch1, Arch2, Arch3, and Arch4 based on ResNet-50 architecture have been proposed. In the first architecture (Arch1), original ResNet-50 architecture has been utilized, however instead of a single FC layer at the end with 1000 outputs or classes, two FC layers have been utilized.

Choosing the number of fully connected (FC) layers in a Convolutional Neural Network (CNN) is a key design decision and depends on several factors, including the complexity of the task, the size and nature of the dataset, and the architecture’s overall design. For tasks with relatively simple decision boundaries (e.g., binary classification or low-resolution image classification), a single fully connected layer may suffice. For more complex tasks, such as multi-class classification on large and diverse datasets, more fully connected layers might be necessary. Other factors may include how large the dataset is, how diverse it is, also size of the feature vector etc. e.g., for a low dimension feature space good results may be obtained at good latency without fully connected layers.

Increasing number of FC layers increases the computational power of neural network, i.e., the network learns more features of the input data. However too much FC layers can also lead to overfitting of the network which affects the generalizing power of the network. So as a tradeoff between complexity and generalizing power of the network, two FC layers have been used. The effectiveness of this approach has been verified through experimentation [48].

The first FC layer has 64 neurons with $L_{1}$ and $L_{2}$ regularization for kernel and biases and ReLU as activation function. The last FC layer has three outputs or classes and Softmax as activation function. Three classes correspond to the three cases, i.e., normal, pneumonia (viral and bacterial), and COVID-19. The model was trained using the transfer learning, in which the last six layers were trained and the remaining layers were frozen. Pretrained network weights corresponding to the ImageNet database were utilized. Similarly, this architecture was trained from scratch as well. The reason that the second last FC layer has 64 neurons is that this value was found to be experimentally optimum. Experiments were also carried out with number of neurons equal to 128 and 32 etc. However, the value of 64 was found to be more suitable.

For designing the second architecture (Arch2), the number of filters of all the layers were reduced by half except the first layer. This is an example of channel pruning. Other approaches are filter and layer pruning. Reducing the number of filters to half reduces the computational complexity of the network significantly while keeping the accuracy at the same level. This has been verified through experimentation (network simulation). One reason is that the full channel ResNet-50 architecture was specifically tailored for ImageNet dataset which has 1000 classes. So, the number of features that need to be learnt are quite high. However, the present work has only 3 classes, so number of features to be learnt are quite low, paving the way for reducing the channels. Also, the first layer of ResNet-50 architecture has been left intact, so the maximum number of low-level features can be extracted from the input images. Another benefit is that it makes the VLSI architecture implementation in radix 2 domain, for example, the number of filters in each layer of ResNet50 are power of 2 and reducing the network complexity while keeping the number of channels power of 2 has benefits in the corresponding VLSI implementation, i.e., efficient adder trees [31], for the additions of convolutions of features coming from input channels, can be incorporated into the VLSI architecture making the layout compact.

Table 12, shows that a light weight CNN architecture such as MobileNet has significantly low number of weights as compared to full channel ResNet-50 CNN but has the same accuracy level. In the same way, reducing channels from the ResNet-50 architecture led to another independent light weight CNN which has almost same accuracy level and hardware implementation benefits.

Reducing the number of channels, makes the network a light weight network for resource constrained applications and the network becomes less prone to overfitting as the computational power of the network has been reduced. On the other hand, layer pruning reduces the depth of the network which may affect negatively the learning capability of the network. So, channel pruning is used instead of filter and layer pruning. The last two FC layers were left unchanged.

In the third architecture (Arch3), the number of filters of all the layers were made equal to 128 except the first layer, which was unchanged, with the last two FC layers unchanged as well. The reason for keeping the first layer unchanged is to extract maximum number of low-level features that will be used to construct high level features by the subsequent layers. Further reduction in number of channels resulted in deteriorated performance of the network so is not considered here. This reduction in channels was further investigated with architecture 4 (Arch4) having number of channels 96, architecture 5 (Arch5) having number of channels 64, architecture 6 (Arch6) having number of channels 32, architecture 7 (Arch7) having number of channels 16 and architecture 8 (Arch8) having number of channels 8.

Another possibility for complexity reduction is to reduce the number of layers. However, since depth of neural network or number of layers is directly linked with the learning capability of neural network the complex features of the input, the number of layers were left unchanged.

### 4.3. Datasets

We created a dataset with a total of 66159 images (normal, pneumonia, and COVID-19) by collecting images from 10 different datasets to train the models with details as shown in Table 2. The aim of using different image sources was so that the model can be trained on a diverse set of images, with different quality, image acquisition hardware, capture protocols, and conditions, in order to train classification models that are generalizable to unseen data from other sources. In addition, we used images from the two datasets as shown in Table 3 to determine the generalizability of the selected models.

**Table 2:** CXR dataset for classification.

Dataset	Images			Used by Ref
	Normal	COVID-19	Pneumonia	
[49]	12544	13861	4245	[2], [50]
[51]	0	468	0	[52]
[53]	0	900	0	[54]
[55]	0	55	0	[56]
[57]	0	243	0	[58]
[59]	10192	3616	1345	[3]
[60]	0	368	0	
[61]	1802	1626	1800	[62]
[63]	1583	576	4273	
[64]	1443	980	4239	
Total Images	27564	22693	15902	

**Table 3:** CXR dataset for model generalization.

Dataset	Images			Used by Ref
	Normal	COVID-19	Pneumonia	
[65]	0	238	0	[56]
[66]	234	221	148	[67]
Total Images	234	459	148	

### 4.4. Pre-processing and data augmentation

As a preprocessing step, histogram equalization was applied. However, it was noticed that the network performance was better without histogram equalization, so the results presented here are without histogram equalization. Similarly, data augmentation, as a preprocessing step was applied which involved random rotation of input image in the range $\left(-10^{o},10^{o}\right)$ and random horizontal flip. However again it was noticed that the generalization accuracy is better without these operations. Also, the fact that the image database has already large number of images, hence data augmentation might not benefit here significantly.

### 4.5. Image classification and model generalization

The proposed architectures were implemented using Python language along with the Tensorflow and Keras libraries. As mentioned in Table 2, CXR images were collected from different databases. The images belonged to the three classes, normal (class 0), pneumonia (class 1), and COVID-19 (class 2). The databases were merged together and the above-mentioned networks were trained. For training, the ten datasets from Table 2 were utilized. The total number of images were approximately 66000, out of which 80% images were used for training and 20% images were used for validation and testing. It is important that the trained model not only performs well on the test set but that it can generalize to other datasets.

Model generalization is significant for the medical images as different datasets at the various hospitals would differ due to the CXR machines, operating procedures, and skills of the technicians. In creating a single dataset from the ten selected datasets, it ensures that the trained model will have better performance on an unseen dataset. In order to further probe into the generalization capability of the networks, another dataset (Table 3) was used for validation or testing purpose. Since this dataset is unbalanced with a greater number of images for class 2, therefore more images for class 0 and 1 were acquired which were not used during the training.

The learning rate used was 10^-3^ and the model was trained for 20 epochs. In the second phase or fine-tuning phase of the training, the network was fine-tuned by training it again for another 10 epochs with learning rate equal to 10^-5^. For Arch1, with transfer learning, last six layers were trained during the first phase and all the layers were trained during fine tuning (second phase). Batch size of 96 images was used. Input CXR images were grayscale images and were rescaled to a dimension of 224×224 pixels to conform to the ResNet-50 architecture.

### 4.6. Explainability

Gradient-weighted Class Activation Mapping (Grad-CAM) [12] is an algorithm for visualizing the features of the input image that the model has used to classify the image as belonging to a particular class. This algorithm involves derivative (gradient) of the output with respect to the last layer of convolution network. In this work, dark red color indicates the region of image that the model is mostly concentrating. Finally, the models were evaluated visually using Grad-CAM algorithm [12].

### 4.7. Performance metrics

In order to evaluate the network architectures, different metrics have been used, such as accuracy, precision, recall, f1 score and confusion matrix. In addition, Receiver Operating Characteristic (ROC) curves and precision-recall curves have also been considered.

Since in this work three classes are involved so it is a multiclass classification problem. Since the dataset is not balanced, therefore relying on accuracy metric [38] for model evaluation may not be a good choice, as it does not tell about the class wise accuracy. Precision, recall and f1 score can evaluate model in terms of class wise performance as shown in Tables 4 and 5.

**Table 4:** Number of parameters involved in the eight architectures.

Architecture	Number of Parameters			Percentage Reduction
	Trainable	Non-trainable	Total	
Arch1	23665923	53120	23719043	0%
Arch2	5981635	26624	6008259	75%
Arch3	2971210	13440	2,984,650	87%
Arch4	1684170	10112	1694282	93%
Arch5	761674	6784	768458	97%
Arch6	203722	3456	207178	99.1%
Arch7	61450	1792	63242	99.7%
Arch8	24490	960	25450	99.9%

**Table 5:** Classification performance of the nine architectures corresponding to the validation data set.

Proposed Architecture	Classification performance			
	Accuracy (%)	Precision (%)	Recall (%)	F1-Score (%)
Arch1-TL	99.7	100	100	100
Arch1-Scratch	99.36	99	99	99
Arch2-Scratch	99.5	100	100	100
Arch3-Scratch	99.5	100	100	100
Arch4-Scratch	99.18	99	99	99
Arch5-Scratch	99.18	99	99	99
Arch6-Scratch	98.72	99	99	99
Arch7-Scratch	97.14	97	97	97
Arch8-Scratch	94.63	95	95	95

ROC curve is a graph showing the performance of a classification model at all classification thresholds. The more the area under the curve approaches unity, the better is the performance of the model. It indicates the separation between the classes and how much easy or difficult it is for a model to misclassify an input image. The more the value of true positive rate (TPR) at low false positive rates (FPR), the better it is. This curve is a plot of TPR vs. FPR. The more is the overlap between the distributions of positive and negative classes, the more difficult it is for a model to separate the classes; hence the model will have reduced performance.

Similarly, precision and recall curve is used as a performance metric for evaluating the performance of the model when the dataset is unbalanced, i.e., one class has a greater number of images than the other classes. Again, the area under the curve approaching unity is taken as a measure of better performance of the model.

## 5. Experimental results

### 5.1. Complexity reduction

Though the training time was also not reduced, but for inference the idea was to reduce the number of weight parameters so that its corresponding VLSI implementation could be made with smaller resources leading to saving in cost and reduction in power consumption etc. Table 4 shows the complexity reduction of the CNN by 75% in case of Arch2 and by 87% in case of Arch3, by 93%.in case of Arch4, by 97% in case of Arch5, by 99.1% in case of Arch6, by 99.7% in case of Arch7 and by 99.9% in case of Arch8.

### 5.2. Classification

Table 5 shows the performance of the proposed CNNs for the combined dataset shown in Table 2. It can be seen that Arch1-TL with transfer learning has the best performance in terms of the performance metrics. It can also be noticed that decreasing the number of channels (features) did not deteriorate the performance significantly.

Tables 6 to 9 show confusion matrices corresponding to the proposed architectures and datasets in Table 3. The left most column indicates the predicted classes, whereas the top row indicates the true classes. Best results correspond to all the off diagonal values being zero and all the diagonal values as nonzero. Table 6 shows that there are five cases (row 2 and column 0) in which normal class has been misclassified as COVID-19 class. Arch1-TL had the best performance followed by Arch2-Scratch.

**Table 6:** Confusion matrix for Arch1-TL.

	Predicted Class			
**Actual Class**	**Classes**	**0**	**1**	**2**
	**0**	447	0	0
	**1**	0	448	0
	**2**	5	0	444

**Table 9:** Confusion matrix for Arch3-Scratch.

	Predicted Class			
**Actual Class**	**Classes**	**0**	**1**	**2**
	**0**	447	0	0
	**1**	0	448	0
	**2**	16	0	433

**Table 7:** Confusion matrix for Arch1-Scratch.

	Predicted Class			
**Actual Class**	**Classes**	**0**	**1**	**2**
	**0**	447	0	0
	**1**	1	447	0
	**2**	25	0	424

**Table 8:** Confusion matrix for Arch2-Scratch.

	Predicted Class			
**Actual Class**	**Classes**	**0**	**1**	**2**
	**0**	447	0	0
	**1**	0	448	0
	**2**	15	0	434

**Fig 2:**
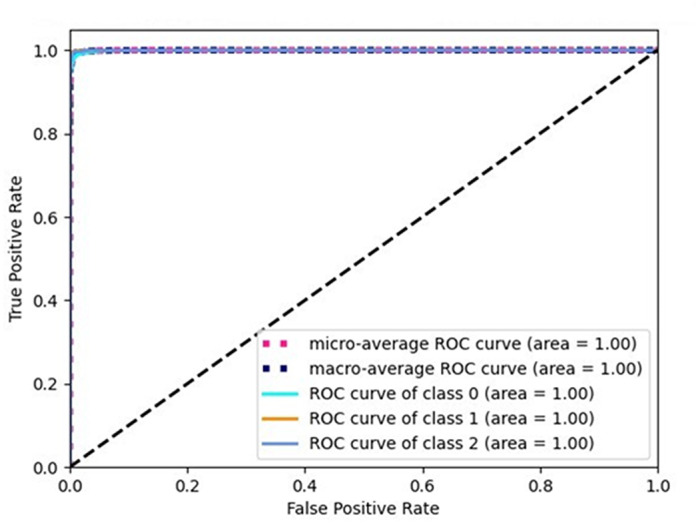
ROC curve for Arch1-TL.

**Fig 3:**
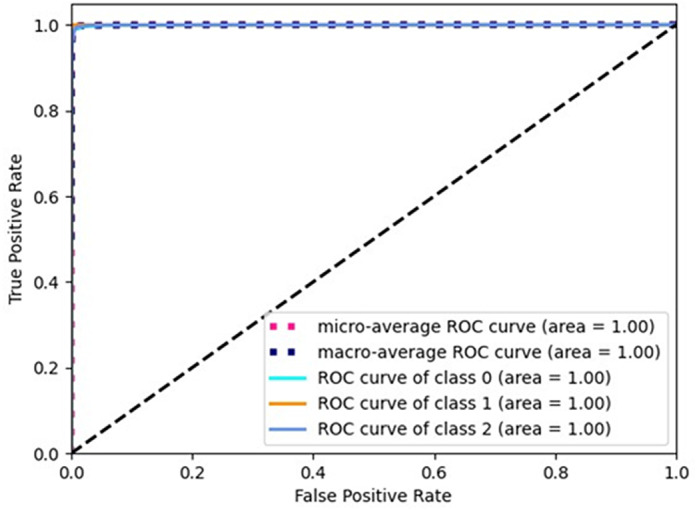
ROC curve for Arch2-Scratch.

**Fig 4:**
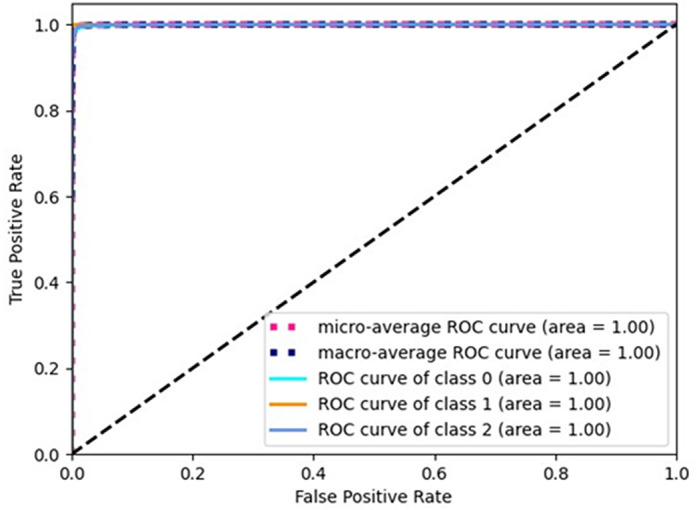
ROC curve for Arch3-Scratch.

**Fig 5:**
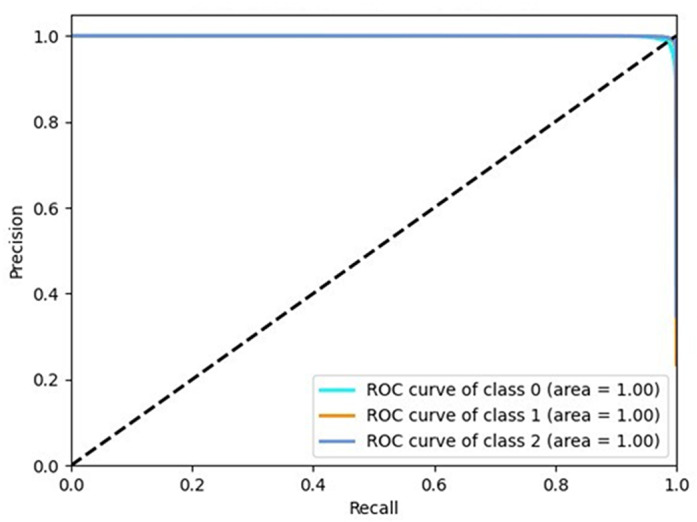
Precision Recall curve for Arch1-TL.

**Fig 6:**
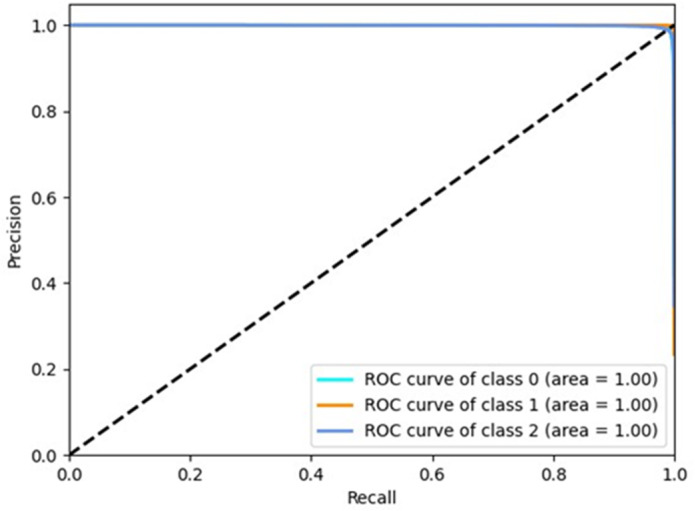
Precision Recall curve for Arch2-Scratch.

**Fig 7:**
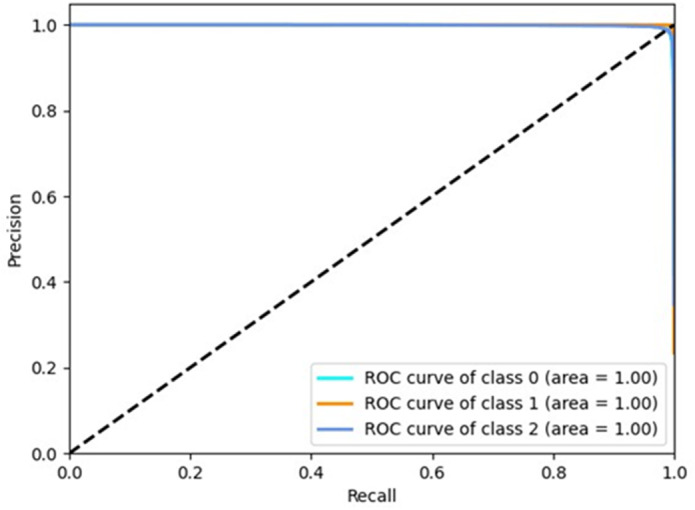
Precision Recall curve for Arch3-Scratch.

Figs 2, 3, and 4 show the ROC curves for the three classes (Normal 0, Pneumonia 1, COVID 2), corresponding to the three selected architectures described in Table 5. As can be seen, the area under the curve is unity in all the cases. Similarly, Figs 5, 6, and 7 shows the precision recall curves for the three classes corresponding to the three architectures. Again, it can be seen that the area under the curves is unity.

### 5.3. Model generalization

Table 10 shows the performance of the proposed architectures on a different dataset (Table 3). Though the performance has decreased compared to Table 5 but the performance degradation is at an acceptance level demonstrating a good generalization capability of the trained model. It is important to notice here that Arch1-Scratch has the least performance. This can be attributed to the fact that full CNN is prone to overfitting and thus reduction of channels or features actually prevents the model from overfitting.

**Table 10:** Classification performance of the three architectures corresponding to another validation data set.

Architecture	Accuracy (%)	Precision (%)	Recall (%)	F1-Score (%)
Arch1-TL	99.62	100	100	100
Arch1-Scratch	98	98	98	98
Arch2-Scratch	98.88	99	99	99
Arch3-Scratch	98.8	99	99	99

### 5.4. Explainability

**Fig 8:**
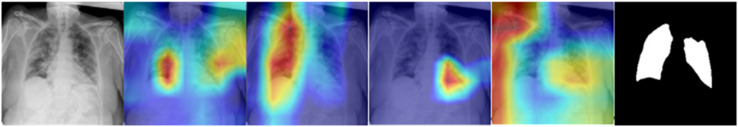
(a) Original grayscale CXR image from the COVID-19 class (b) Grad-CAM result for Arch1-TL (c) Grad-CAM result for Arch1-Scratch (d) Grad-CAM result for Arch2-Scratch (e) Grad-CAM result for Arch3-Scratch (f) Ground truth image.

**Fig 9:**
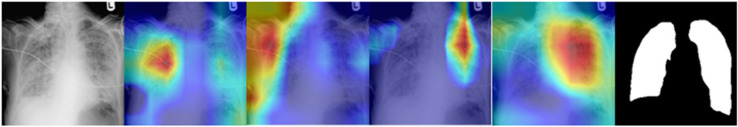
(a) Original grayscale CXR image from the COVID-19 class (b) Grad-CAM result for Arch1-TL (c) Grad-CAM result for Arch1-Scratch (d) Grad-CAM result for Arch2-Scratch (e) Grad-CAM result for Arch3-Scratch (f) Ground truth image.

**Fig 10:**
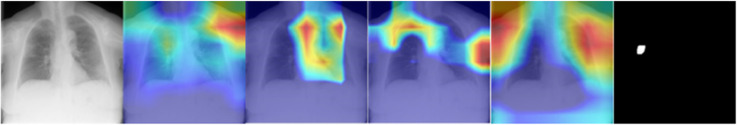
(a) Original grayscale CXR image from the COVID-19 class (b) Grad-CAM result for Arch1-TL (c) Grad-CAM result for Arch1-Scratch (d) Grad-CAM result for Arch2-Scratch (e) Grad-CAM result for Arch3-Scratch (f) Ground truth image.

Three X-ray images from the validation data set were chosen to evaluate the efficacy of the Grad-CAM technique. The Grad-CAM results corresponding to the proposed architectures were generated and are shown in Figs 8–10 along with the corresponding ground truth images.

### 5.5. Performance comparison with state-of-the-art models

In this Section, we provide a comparison with the recent results for severity grading of COVID-19 images. In [31], authors have presented a comparative analysis of CNN architectures, such as ResNet-50, ResNet-101, DenseNet121, DenseNet169 and InceptionV3, for COVID-19 classification from three classes, COVID-19, Non-COVID-19 and Normal. They reported the performance of these networks as satisfactory in terms of performance evaluation metrics such as accuracy, precision and recall. They also reported ResNet-101 CNN as superior to all these networks achieving 96% accuracy, precision and recall. In [68], authors have presented a unique detection and classification approach, DCCNet for quick diagnosis of COVID-19 using CXR images of patients. Authors proposed an approach which is hybridization of CNN and histogram of oriented gradients. The approach was evaluated on different CXR image datasets. They reported 99.67% testing accuracy. In [69], authors initially investigated and compared eleven existing CNN models, and found MobileNetV2 CNN as promising candidate for further modification to achieve high and acceptable accuracy for COVID-19 detection and classification. Their resulting proposed model achieved 98% classification accuracy which was better than the existing methods. In [70], multiple state-of-the-art CNNs such as DenseNet201, ResNet-50V2 and InceptionV3 have been investigated and then combined using a new method of weighted average ensembling to predict a class value. The authors reported testing accuracy of 95.7% and recall and f1-score as 98% and 96.2% respectively. In [71], authors proposed a CNN known as CoroDet for two class classification (COVID-19 and Normal), three class classification (COVID-19, Normal and viral Pneumonia) and four class classification (COVID-19, Normal, viral Pneumonia and bacterial Pneumonia). They reported 99.1% testing accuracy for two class classification, 94.2% testing accuracy for three class classification and testing accuracy of 91.2% for four class classification. In [72], four popular CNNs including ResNet-18, ResNet-50, SqueezeNet and DenseNet121 were trained and evaluated on CXR images for the purpose of COVID-19 detection and classification. The authors reported that most of the networks achieved sensitivity rate of 98% (+3%, -3%) and specificity rate of around 90%, where sensitivity rate was defined as number of correctly predicted images as COVID-19 divided by total number of COVID-19 images and specificity was defined as number of correctly predicted non-COVID-19 images divided by total number of non-COVID-19 images. Similarly, in [73], authors trained and evaluated different popular CNNs including VGG16 and MobileNet for the purpose of COVID-19 detection and classification. They reported that VGG16 and MobileNet outperformed all other networks while achieving testing accuracy of 98.28%. In addition, they reported that VGG16 outperformed all other models in COVID-19 detection with an accuracy, F1 score, precision, specificity, and sensitivity of 98.72%, 97.59%, 96.43%, 98.70%, and 98.78%, respectively. In [74–79] authors have investigated classification of COVID-19 using derivative of ResNet50 architecture.

The model performance comparison is provided in Table 11. The last three rows highlight that there is an insignificant decrease in the model performance corresponding to a reduction in the model parameters by 75%, 87% and 93% for the proposed Arch2-Scratch, and Arch3-Scratch and Arch4-Scratch respectively.

**Table 11:** Comparison of the proposed architectures with the state-of-the-art for COVID-19 classification.

Ref.	Model	Testing Accuracy	Avg. Precision	Avg. Recall	Avg. F1 score	Number of parameters
[31]	ResNet-101	96%	96%	96%	96%	25040899
[68]	Deep learning	99.1%	99%	99%	99%	–
[69]	Deep learning algorithms	98%	97%	98%	97%	–
[70]	CNN	95.7%	–	98%	96.2%	–
[71]	Deep CNN	99.1%	98.5%	97.5%	97.5%	–
[72]	Deep CNN	–	90%	98%	–	–
[73]	CNN	98.7%	96.4%	98.7%	97.5%	–
Proposed Arch1-TL	CNN	99.7%	100%	100%	100%	23719043
Proposed Arch1-Scratch	CNN	99.36%	99%	99%	99%	23719043
Proposed Arch2-Scratch	CNN	99.5%	100%	100%	100%	6008259
Proposed Arch3-Scratch	CNN	99.18%	99%	99%	99%	768003

Depthwise separable convolution (DSC) is a low complexity version of standard convolution in which a 1x1 point convolution is applied along the depth, i.e., along the channels and the result is added together. This gives one channel at the output. This process is repeated for the number of output channels to obtain the required number of output channels [39].

The authors of the present work have implemented DSC in ResNet50 architecture and trained the model from scratch. Similarly, light weight convolution networks such as MobileNet [80], NASNetMobile [81] and Xception [82] have also been investigated and implemented for comparison purpose, using the same dataset, and the resultant accuracy and complexity are mentioned in Table 12. Results have shown that the proposed scheme has reduced complexity than the state-of-the-art low complexity networks with accuracy at the same level.

**Table 12:** Complexity and performance metrics of the state-of-the-art low complexity networks.

	Testing Accuracy (%)	Number of parameters
ResNet50 with DSCV	99.26	4598787
MobileNet	99.22	2542856
NASNetMobile	99.4	5289978
Xception	97.5	22855952

Table 13 shows the performance of the proposed technique on CIFAR-10 dataset [83]. The validity of the proposed methodology is further evident from Table 13, as the full channel Arch1 and proposed reduced channel architectures, i.e., Arch1-Scratch, Arch2-Scratch, Arch3-Scratch and Arch4-Scratch have almost the same accuracy level.

**Table 13:** Model testing on CIFAR-10 dataset.

Architecture	Accuracy (%)
Arch1-TL	94
Arch1-Scratch	90
Arch2-Scratch	90
Arch3-Scratch	90
Arch4-Scratch	89
Arch5-Scratch	85
MobileNet	88.4

### 5.6. Theoretical VLSI architecture

**Fig 11:**
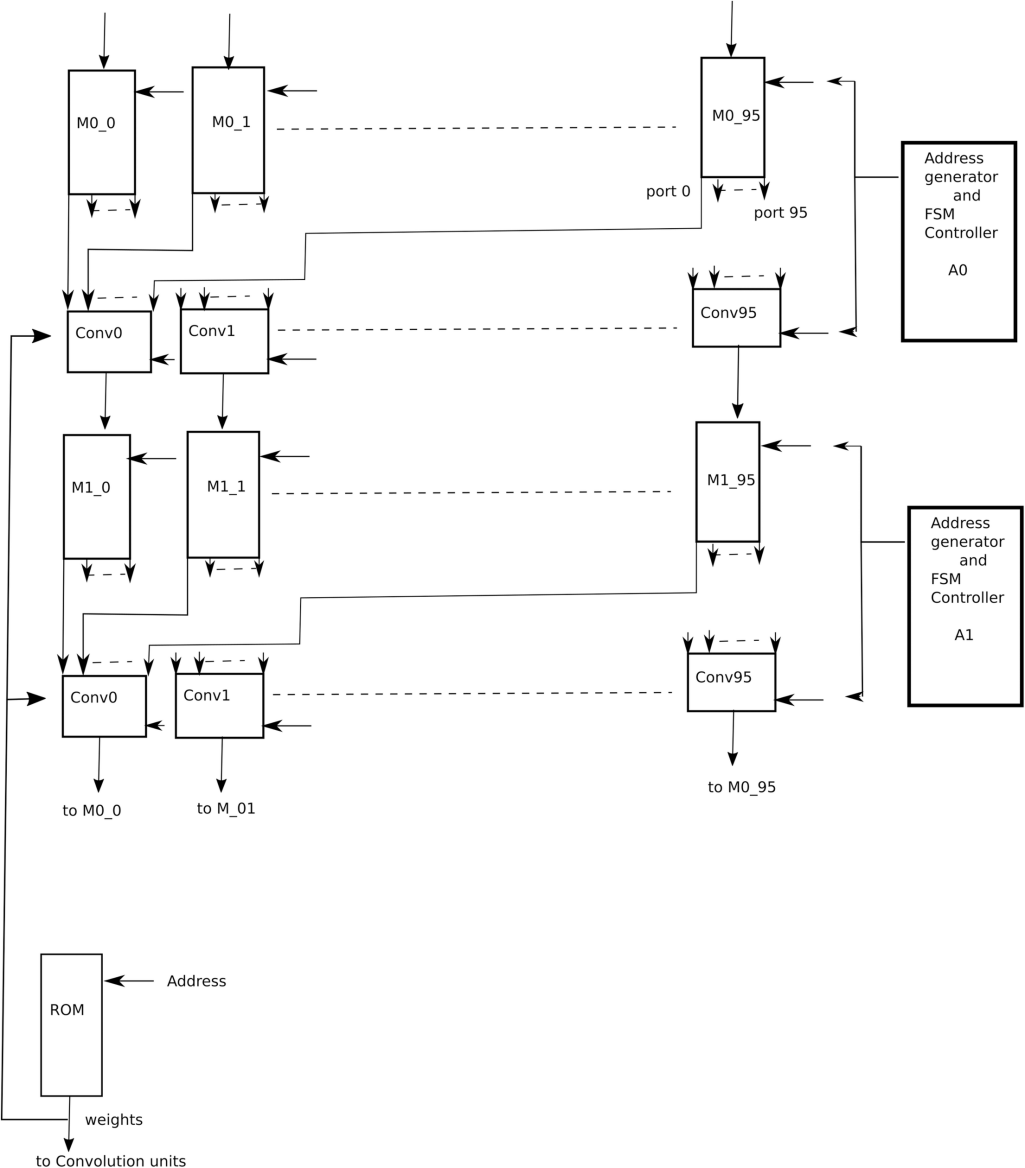
A parallel VLSI architecture.

**Fig 12:**
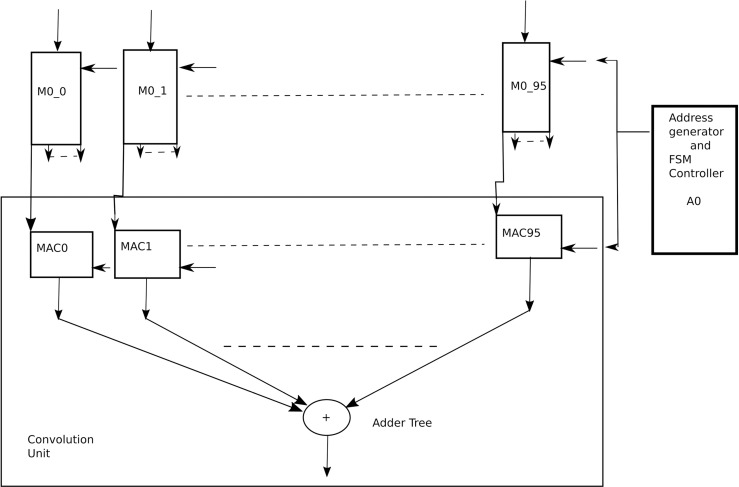
A Convolution unit.

**Fig 13:**
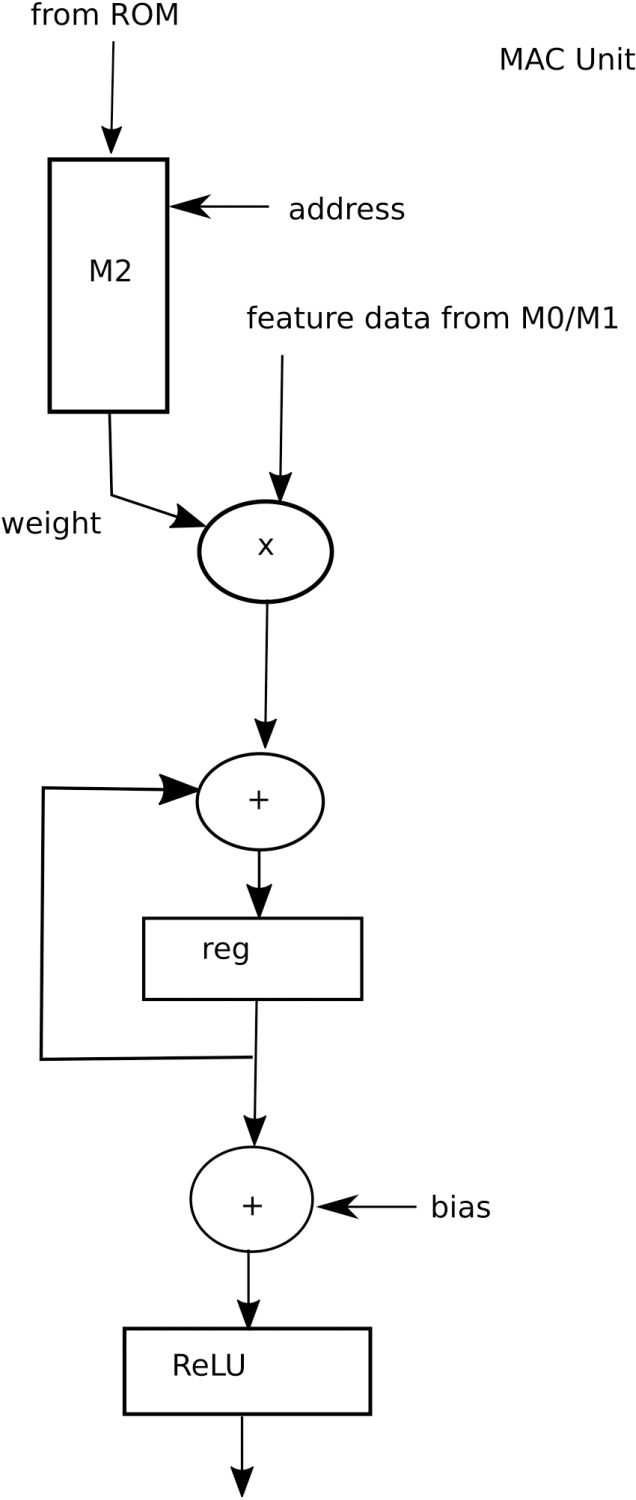
A Multiply and Accumulate (MAC) unit.

After considering results from Tables 4, 5, 10, 11, 12 and 13, it was decided to present a VLSI architecture for two intermediate layers of architecture 4 (Arch4), k and k + 1, as shown in Fig 11, which will be reused for the remaining layers of the CNN. The reason for choosing Arch4 is that it has almost the same accuracy level as that of full channel architecture, however at much reduced complexity level. Each layer, as shown in Fig 11, has 96 input channels and 96 output channels. Considering the case for kernel size of 3x3, there are 96x96 filters in each layer, each of size 3x3, and each weight coefficient is 32-bits wide for the full precision. As shown in Table 4, there are 1,694,282 parameters, which will be stored in a Read only memory (ROM) of size 2097152 x 32-bits, where the 2097152 corresponds to 2x1024x1024 locations of ROM which corresponds to 21 address bits. The weights are stored in row major order, one filter after the other. Each of the two layers has 96 convolution units, Conv0 to Conv95. Each convolution unit, as shown in Fig 12, has 96 MAC (multiply and accumulate) units and activation units (ReLU). Each MAC unit, as shown in Fig 13, is responsible for the computation of a convolution operation serially. At the input of the architecture is 96 Random access memories (RAMs), M0_0 to M0_95. Each of these RAMs has 96 read ports and one write port. The size of these RAMs is 64K x 32-bits to store the 224x224 sized features in row major order. At the output of layer k are again 96 RAMs, M1_0 to M1_95 each of size 8x32-bits to store the intermediate results of convolution and activation from layer k and inputting them into layer k + 1 convolution units. Each of these RAMs has 96 read ports and one write port.

Each of the MAC units in layer k has associated RAM, M2, of size 16x32 bits to store the 3x3 weight coefficients. There is a common address generator unit A0 for layer k that computes the addresses for memory M2 and memories M0. Similarly, for layer k + 1, there is an address generator unit A1 which computes addresses for memory M2 of layer k + 1 and memories M1.

At the start, another address generator A3, will read the ROM and start writing the corresponding weight coefficients in the corresponding RAMs M2 of both layers. Assuming all features from previous layer k-1, have been written to the memories M0. The address generator A0 will generate address for first coefficient in memories M2 in layer k and the first coefficient in memories M0. With each cycle, an address will be generated and the MAC unit will execute the MAC operation. It is to be noted here, that this architecture is parallel architecture, and all the MAC units of all the convolution units will execute their corresponding operation in each clock cycle.

The writing of M2 memories of both layers from the ROM takes 96x96x9 + 96x96, i.e., 92160 clock cycles. Where 96x96 implies that each layer has 96x96 filters which are 9216. Each filter has 9 weight coefficients and one bias value. After next 10 clock cycles one convolution operation by each convolution unit will be completed. Assuming features to be zero padded in order to maintain the dimension of the feature, there are 224x224 convolution operations which takes 224x224x10, i.e., 501760 clock cycles. When one convolution operation finishes, it goes through ReLU unit and the corresponding result is saved in the corresponding memories M1. After 7 rows of features from the convolution layer k have been written to memories M1, the convolution units of layer k + 1 will have enough data to start computing the convolutions. So their convolution operations will commence in parallel with layer k. As the features from layer k-1, which were stored in M0 memories are no longer required, so the output features form layer k + 1 will be written there. As mentioned before, after 501760 clock cycles, the convolution units of layer k will be free so they will start computing convolutions for layer k + 2. This process will get repeated until all the layers have been processed. This way, maximum hardware utilization is achieved as no unit is idle. All the hardware is busy till the end of the processing.

The reason for keeping memories M1 of size 7x224 is that when one row of features from layer k gets processed, it is no longer needed so it will be replaced by the next convolution, e.g., first row of features from layer k will be replaced by the eighth feature and so on.

Total clock cycles required by the two layers to complete the operations are 92160 + 501760, i.e., 593920 clock cycles. The ResNet-50 architecture has 50 convolution layers, so the total clock cycles required are 25x593920 which are 14848000.

The aim is to render the inference of input image in 2 seconds, i.e., on the spot in real time. Hence the VLSI architecture requires at most 7.5 MHz clock frequency to carry out all the computations. It is also important to mention here that this estimate is the maximum estimate. There are some layers which require convolution kernel of size 1x1. Hence the actual frequency requirement will be lesser, and not very difficult to achieve in today’s ultrascale FPGAs and ASIC technologies.

One deficiency in this architecture is that it has 96 channels per layer and 96 is not power of two, hence a compact radix adder tree cannot be used however the problem arises at the last layer of adder tree, for example, for adding 96 convolutions together, the first layer of adder tree has 48 two input adders, then next layer has 24 adders, then 12 adders, then 6 and 3 adders. Hence two more adders will be required to yield the sum requiring 7 levels.

## 6. Conclusion and further work

In this paper, we have proposed a simplified approach for complexity reduction by reducing the number of channels of the DNN to significantly reduce the number of parameters. We evaluated the effect on the performance of a deep neural network due to its size reduction. We have implemented this by reducing the number of channels for the ResNet-50 model but the techniques can be similarly applied to other neural network architectures.

In order to evaluate the model performance, we selected the multiclass classification problem of COVID-19 classification. It is difficult for the radiologists to differentiate between COVID-19 and pneumonia which appear quite similar in chest X-ray (CXR) images. Deep Neural Networks (DNN) have been extensively used to automatically learn the differentiating features and classify images but the understandability and trust in the model’s predictions is lacking which can hinder its use in clinical practice. In this study, we propose a reduced complexity ResNet-50 model for multiclass classification and demonstrate through the use of explainability technique, Grad-CAM that the visualisation of the contributing features and regions in the CXR images can help the radiologists to verify their own analysis against the model’s prediction, resulting in better diagnosis and patient’s health management. Overall, architecture with transfer learning, i.e., Arch1-TL has best performance, however has also high computational complexity associated. This work has suggested ways for reducing the computational complexity efficiently. We have also presented a theoretical VLSI architecture for architecture 4 (Arch4) as Arch4 has almost the same performance however at much reduced complexity, i.e., number of weights or filter channels.

In future work, we will investigate the complexity reduction and the corresponding model performance of few other selected models. We would also like to research Very Large-Scale Integration (VLSI) implementations of the recent high performing DNN architectures.

## Supporting Information

S1 Appendix Supporting information file. (DOCX)
