# Supplementary material for: Performance evaluation of reduced complexity deep neural networks
Source: PLoS One. 2025 Mar 20;20(3):e0319859. doi: 10.1371/journal.pone.0319859 (PMC11925470; doi:10.1371/journal.pone.0319859)
Supplement: S1 Appendix — (DOCX) [file pone.0319859.s001.docx]

**Supporting Information File**

1. The URLs to retrieve the minimal dataset for **classification** (Table 2) are:
2. https://github.com/ieee8023/covid-chestxray-dataset/tree/master
3. https://github.com/ml-workgroup/covid-19-image-repository
4. https://data.mendeley.com/datasets/xztwjmktrg/3
5. https://data.mendeley.com/datasets/dvntn9yhd2/1
6. The URLs to retrieve the minimal dataset for **model generalization** (Table 3) are:
7. https://data.mendeley.com/datasets/fvk7h5dg2p/3
